# Avulsion Fracture of the Anterior Superior Iliac Spine in a Young Athlete Detected by Point-Of-Care Ultrasound 

**DOI:** 10.24908/pocus.v7i1.15096

**Published:** 2022-04-21

**Authors:** Takaaki Mori, Takateru Ihara, Osamu Nomura

**Affiliations:** 1 Division of Pediatric Emergency Medicine, Department of Pediatric Emergency and Critical Care Medicine, Tokyo Metropolitan Children's Medical Center Fuchu Tokyo Japan; 2 Department of Emergency and Disaster Medicine, Hirosaki University Hirosaki, Aomori Japan

**Keywords:** ultrasonography, pelvic fracture, child, avulsion fracture

## Abstract

Pelvic avulsion fractures (PAFs) are common in adolescents. X-ray is commonly used to diagnose PAF, but the use of point-of-care ultrasound (POCUS) for this purpose in pediatric emergency departments has yet to be published. We reported herein a pediatric case of anterior superior iliac spine (ASIS) avulsion fracture detected by POCUS. A 14-year-old male patient visited our emergency department for groin pain he experienced during a game of baseball. POCUS of the right ilium demonstrated a hyperechoic structure anterolaterally displaced towards the ASIS, suggesting an ASIS avulsion fracture. X-ray of the pelvis confirmed the findings and led to the diagnosis of ASIS avulsion fracture.

## Introduction

Pelvic avulsion fractures (PAFs) are rare and specific to adolescents and young athletes 1, 2. Owing to their stage of musculoskeletal development, forceful contractions of muscles or tendons during sports activities frequently cause a PAF to occur in any of four anatomical sites, including the iliac crest, anterior superior iliac spine (ASIS), anterior inferior iliac spine (AIIS), and ischial tuberosity 1, 2. X-ray is normally used to diagnose PAFs, but the condition can be misdiagnosed if the fragments of the fractured bones are small 3. Computed tomography (CT) or magnetic resonance imaging (MRI) may also be used but they are expensive to perform. Point-of-care ultrasound (POCUS) is an alternative modality for diagnosing long bone fractures or ruptured tendons,^4^, ^5^ but the reports of its use for this purpose in the pediatric emergency care setting are scarce. We herein reported a pediatric case of ASIS avulsion fracture detected by pediatric emergency physicians using POCUS.

## Case Report

A previously healthy, 14-year-old male patient visited our emergency department for right groin pain which occurred when he suddenly changed course while running during a game of baseball. The pain prevented him from walking. He denied paresthesia or testicular pain. His vital signs were appropriate for his age. Physical examination revealed tenderness in the ASIS area but denied tenderness in the iliac crest or femoral head. His right leg was slightly flexed, and the range of motion of his right pelvic joint was limited because of the pain. 

**Figure 1:**
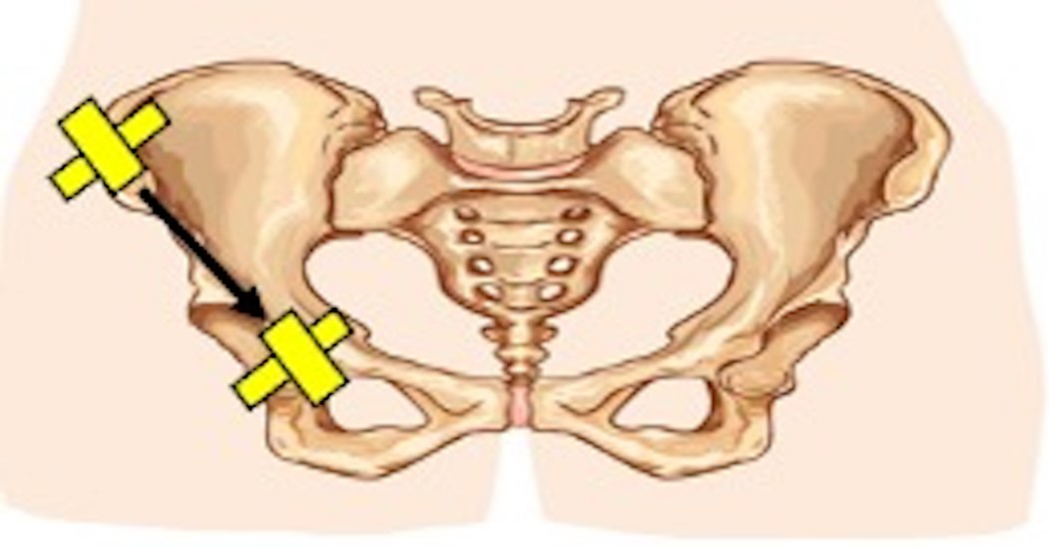
POCUS protocol. A high-frequency linear transducer (8-13 MHz) was placed transversely on the pelvis to scan the area from the iliac crest to the ischium.

**Figure 2:**
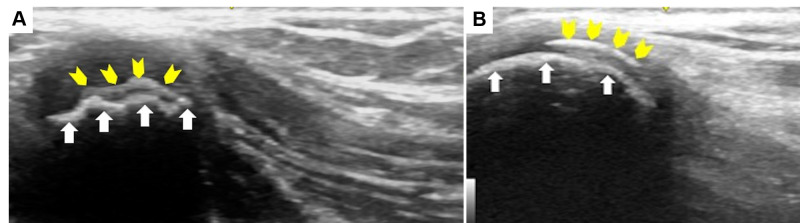
POCUS findings (ASIS view). Transverse (A) and longitudinal view (B) of the ASIS showed two hyperechoic structures with acoustic shadows. POCUS visualized ASIS as an uneven structure (indicated by the arrow), and the apophysis as a curved structure (indicated by the arrow head). The apophysis normally overlies the ASIS when not displaced.

**Figure 3:**
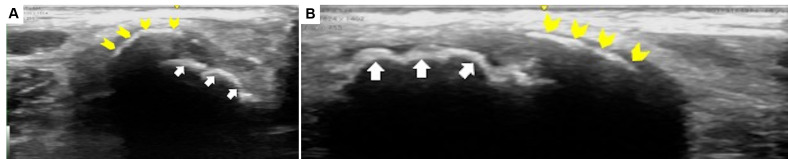
POCUS findings (ASIS view). Transverse (A) and longitudinal view (B) of the ASIS showed two hyperechoic structures with acoustic shadows. The uneven structure is the ASIS (arrow), and the curved structure is the apophysis (arrow head). In the present patient, the apophysis was anterolaterally displaced.

An attending pediatric emergency physician with five years’ experience using pediatric POCUS performed a scan using LOGIQ ^TM^ e (GE Healthcare, Japan) with a high-frequency linear transducer (8-13 MHz). The patient was placed in a supine position, and a transducer was placed transversely and longitudinally from the iliac crest to the ischium (Figure 1). POCUS normally allows visualization of the ASIS and its apophysis as hyperechoic structures with an acoustic shadow, with the apophysis overlying the ASIS (Figure 2). In the present patient, the apophysis appeared hyperechoic with an acoustic shadow on the anterolateral side of the ASIS with an anterior and lateral displacement of 3.6 mm and 3.8 mm, respectively, suggesting an ASIS avulsion fracture (Figure 3).

**Figure 4:**
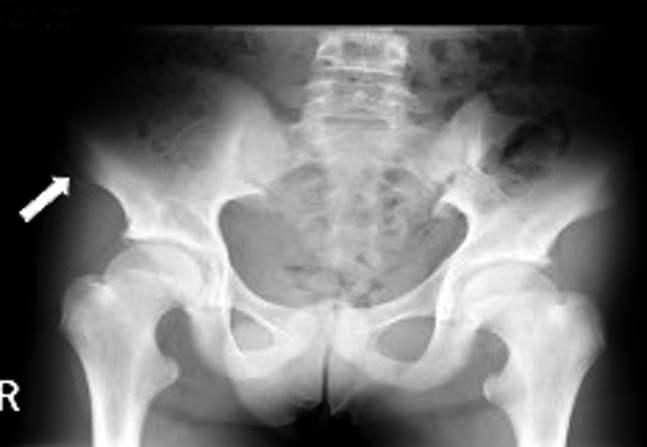
X-ray of the pelvis. A slightly displaced radiopaque structure was detected on the lateral side of the ASIS, suggesting an ASIS avulsion fracture (arrow).

Based on the findings, an ASIS avulsion fracture was suspected. X-ray of the pelvis confirmed the diagnosis of ASIS avulsion fracture (Figure 4). The displacement of the fragment was calculated for surgical fixation but was found to be 3.8 mm (< 20 mm), allowing conservative management. The patient was discharged with conservative treatment with non-weight bearing and had no complications at one month after discharge. 

## Discussion

To the best of our knowledge, the present report is the first to describe the use of POCUS to identify an ASIS avulsion fracture in an adolescent in the pediatric emergency department setting. Reports of ultrasound use in diagnosing PAFs are scarce. A previous case report from a rehabilitation department showed the utility of ultrasound for detecting an ASIS avulsion fracture in adolescent male although the technique was performed after a pelvic X‑ray finding led to suspicion of a fracture 6. A previous case-series study demonstrated that ultrasound performed by a radiologist was useful in detecting ASIS and AIIS avulsion fractures 7. Furthermore, several studies demonstrated the usefulness of ultrasound used by orthopedists evaluating pelvic stability in adult patients with traumatic pelvic fractures 8. However, there are no previous studies of POCUS use by pediatric emergency physicians to detect PAF.

PAF comprises 4% of pelvic fractures and only 1.4% of all fractures. It mainly occurs in adolescents with a mean age of around 14 years^1^, ^2^ most commonly at one of four sites, including the origin of the rectus femoris at the anterior inferior iliac spine (AIIS); the sartorius; the tensor fasciae latae at the ASIS; the hamstring at the ischial tuberosity; and the tensor fasciae latae at the iliac crest 1. The frequency of avulsion fractures at the ischial tuberosity, ASIS, AIIS, and iliac crest is 31.9%, 33.9%, 25.2%, and 5.9%, respectively 2, 9. Both direct forces, such as a hard tackle, and indirect forces, such as those involved in kicking a ball, running or sprinting may cause an ASIS fracture 9, 10. However, lack of awareness about PAFs often leads to its misdiagnosis as muscle strain, ligament injury or apophysitis 2. A previous case-series study discussed five cases of ASIS fracture that were initially misdiagnosed only by physical examination as muscle strain 3. Therefore, when adolescent patients present with pelvic pain, especially engaging in specific, sports-related movements like those mentioned above, POCUS can be effective in differentiating PAF from other pathologies. 

Treatment of PAFs is mainly conservative, involving non-weight bearing and analgesics. Surgery is indicated only if the displacement of the fracture is > 20 mm 2. A retrospective study demonstrated that 97% of PAF patients were treated conservatively 9. A meta-analysis showed no statistically significant difference in clinical outcomes in PAFs without a severe displacement > 15 mm 11.

X-ray is normally used to diagnose PAF and is useful as long as the site of injury is first correctly identified. A retrospective study of 228 cases of pelvic apophyseal avulsion fractures in adolescents revealed that x-ray was able to diagnose 99% of PAF cases correctly and to identify the fracture displacement and fracture accurately 9, demonstrating a level of usefulness comparable to that of CT or MRI, which are only performed for PAF diagnosis when the radiographic findings are in doubt.

POCUS has the potential to be a screening method for diagnosing PAFs in adolescents. First, understanding of the unique mechanism of injury and the PAF presentations will help the treating physicians focus on scanning the most likely sites of PAF occurrence. POCUS allows these areas to be scanned, increasing the likelihood of identifying the site of injury rapidly. Second, as discussed by Martinoli et al. 12, unlike x-ray, POCUS can demonstrate the anatomical details of the pelvis, including the bones, muscles, and tendons, to help differentiate of PAF from other pathologies, enabling PAF to be differentiated from muscle or tendon injuries. Furthermore, POCUS enables a correct measurement of the displacement size, clarifying the need for surgical fixation, while avoiding CT and MRI. While the quality of its findings are operator-dependent with adult avulsion fractures of the ischial tuberosity sometimes being misidentified as a hematoma 13, and a certain amount of training is required to obtain an adequate image 14, pediatric emergency physicians can be trained to use POCUS as an useful modality to diagnose PAFs in the same way they use it to diagnose long bone fractures 15. Although further research is needed, the current report demonstrated that a pediatric emergency physician can readily use POCUS to diagnose an ASIS avulsion fracture.

## Conflicts of Interest

Takaaki Mori, Takateru Ihara, and Osamu Nomura have no financial relationships relevant of this article to disclose.

## Ethical consideration

Written informed consent to publish details of this case was obtained from the patient. 
